# Rational design of on-chip gold plasmonic nanoparticles towards ctDNA screening

**DOI:** 10.1038/s41598-021-93207-7

**Published:** 2021-07-09

**Authors:** Amogha Tadimety, Ziqian Wu, John H. Molinski, Russell Beckerman, Congran Jin, Lauren Zhang, Timothy J. Palinski, John X. J. Zhang

**Affiliations:** 1 Thayer School of Engineering, Dartmouth College, Hanover, 03755 USA; 2 The Lawrenceville School, Lawrenceville, 08648 USA

**Keywords:** Tumour biomarkers, Nanoscale devices, Techniques and instrumentation

## Abstract

This paper demonstrates the design, synthesis, simulation, and testing of three distinct geometries of plasmonic gold nanoparticles for on-chip DNA screening towards liquid biopsy. By employing a seed-mediated growth method, we have synthesized gold nanospheres, nanorods, and nanobipyramids. In parallel, we developed numerical simulations to understand the effects of nanoparticle geometry on the resonance features and refractive index sensitivity. Both experimental and simulation results were compared through a series of studies including in-solution and on-chip tests. We have thoroughly characterized the impact of nanoparticle geometry on the sensitivity to circulating tumor DNA, with immediate implications for liquid biopsy. The results agree well with theoretical predictions and simulations, including both bulk refractive index sensitivity and thin film sensitivity. Importantly, this work quantitatively establishes the link between nanoparticle geometry and efficacy in detecting rare circulating biomarkers. The nanobipyramids provided the highest sensitivity, approximately doubling the sensitivity compared to nanorods. To the best of our knowledge this is the first report carrying through geometric effects of simulation to clinically relevant biosensing. We put forth here synthesis and testing of three nanoparticle geometries, and a framework for both experimental and theoretical validation of plasmonic sensitivities towards liquid biopsy.

## Introduction

Circulating tumor DNA (ctDNA) is of significant interest in the fields of cancer monitoring, screening, and diagnosis^1–4^. ctDNA is shed off of the tumor into the bloodstream with a very short half-life, and thus could be a good indicator of genetic and epigenetic changes within the tumor^5^. Typical methods of ctDNA analysis involve nucleic acid amplification and sequencing, due to low ctDNA concentration in complex biofluids. This amplification process takes time, necessitates specialized reagents, and requires significant instrumentation, making real-time detection challenging. A number of novel nanobiosensors have been developed for detection of ctDNA, including electrochemical, resistive, colorimetric, and microfluidic sensors^6, 7^. Plasmonic sensors provide a promising alternative to amplification-based nucleic acid detection strategies because they have the potential to directly transduce DNA binding events.

Surface plasmon resonance is the collective oscillation of electrons at a metal–dielectric interface. This phenomenon has been used for measuring and monitoring biochemical interactions because of the local change in refractive index at the surface of the sensor^8^. The resonance is associated with highly sensitive electric fields which can measure changes in the local environment due to minute refractive index changes. As such, this process does not require fluorescence or other readout methods to amplify signals^9^ and can provide simpler sensing workflows with fewer chemical steps^10, 11^.

Metallic nanoparticles have long been used for biosensing due to their applications for colorimetric detection, fluorescence enhancement, and electrochemical detection^12^. Other groups have exploited their plasmonic properties for surface enhanced Raman spectroscopy (SERS), plasmonic imaging, and bulk refractive index sensing^13–15^. These nanoparticles can also support Localized Surface Plasmon Resonance (LSPR) at their surface, and have been extensively investigated for sensing applications^11, 16^. The modes are governed by the material, geometry, and arrangement of the nanoparticles and change with the surrounding dielectric environment^17– 19^. It is well understood that plasmonic properties of nanoparticles are enhanced by sharp corners, which allow for plasmonic hotspots^14, 20–23^. Thus, more interesting nanoparticle geometries may allow for plasmonic enhancement when applied to biomolecular sensing. These other geometries will also have uniquely shaped resonance spectra that may have higher figures of merit or sensitivity to binding events^16^.

Our prior work has demonstrated significant progress in fabrication of novel geometries of nanoparticles, including porous and hollow spheres, rods, and ellipsoids^24–27, 28, 29^. Compared to existing nanoparticles, these particles have been demonstrated to provide significant improvements in drug loading, dye adsorption, and cell targeting. For plasmonic sensing, it is expected given the literature that particles with such novel geometries could provide sensitivity enhancement and an interesting system to study LSPR biosensing^30^. This work is novel compared to the existing literature because of the thorough comparison of plasmonic simulation and experiment through to preclinical application. This provides a framework for rational design of nanoparticles for biomarker-specific plasmonic screening.

This paper thoroughly studies the effects of gold nanoparticle geometry on sensitivity towards ctDNA screening in liquid biopsy. Three geometries of solid gold nanoparticles are synthesized: nanospheres, nanorods, and nanobipyramids. Previous research has demonstrated the improved electromagnetic enhancement and near-field sensitivity in nanoparticles with sharp tips. Yet, a quantitative comparison of these different nanoparticle geometries in a realistic biosensing scenario has been lacking until now. In this work, we systematically study the effects of geometry both in bulk and sequence-specific sensing scenarios. In parallel numerical electromagnetic simulations are developed to theoretically understand the effects of geometry on plasmonic resonance and sensitivity. The synthesized particles are tested to understand their bulk refractive index sensitivity and their ctDNA screening sensitivity. The nanoparticles are well dispersed and randomly oriented on the chip to avoid the effect of near-field coupling, so that ensembles of isolated nanoparticles are measured during the test. At each stage the experimental and simulation results were compared, with good agreement. Together this paper demonstrates a workflow for rational design of solid gold nanoparticles to improve sensitivity for ctDNA screening.

## Methods

### Overall paper workflow

**Figure 1:**
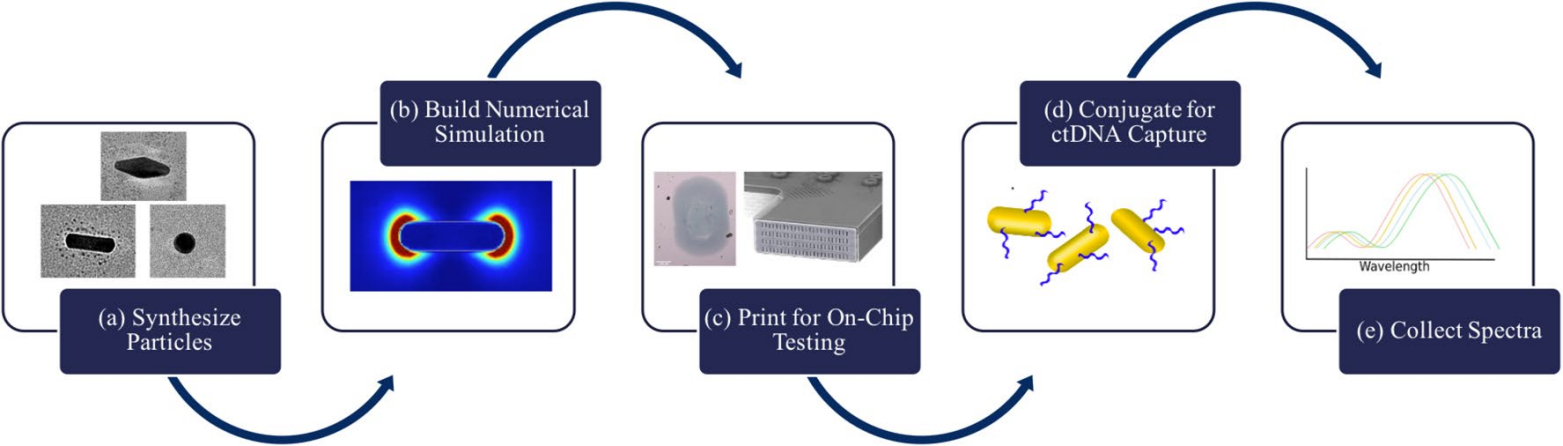
Overall study workflow*.* In this paper, the effects of nanoparticle geometry on plasmonic sensitivity are probed in the context of ctDNA liquid biopsy. First, (**a**) synthesis of gold nanoparticles and (**b**) development of numerical simulations with matching geometry. Then (**c**) dispersion of particles on-chip using microfluidic printing for (**d**) ctDNA capture and (**e**) comparison of the spectral sensing results to the simulation. This allows for an evaluation of the biosensing performance of the synthesized particles and how well it compares to simulation.

Figure 1 demonstrates the workflow for the study. Gold nanoparticles were synthesized and numerical simulations developed with the same geometry. Then, in parallel, the synthesized particles were tested for response to changes in bulk refractive index and ctDNA screening sensitivity. Experimental results of these tests were compared to simulation.

### Nanoparticle synthesis and characterization

Gold nanorods, nanospheres, and nanobipyramids were synthesized in solution. Our protocols were developed with reference to seminal work forming the basis of nanoparticle synthesis^31, 32^. Nanospheres were synthesized by reducing the chloroauric acid solution by heating with the employment of sodium citrate^31^. The nanorods and nanobipyramids were synthesized using a seed mediated method^33, 34^. The process started with gold seed solution which contains small gold nanoparticles. The seed solution was then mixed with a plate solution to slowly grow gold around the existed seeds for target geometries.

All nanoparticles were characterized using transmission electron microscopy (FEI Tecnai F20ST FEG TEM). Particles were bound to TEM grids in the laboratory, and then systematically imaged at a range of magnifications using transmission electron microscopy. Hundreds of each geometry of nanoparticle were imaged to allow for optimal nanoparticle size distributions.

**Figure 2:**
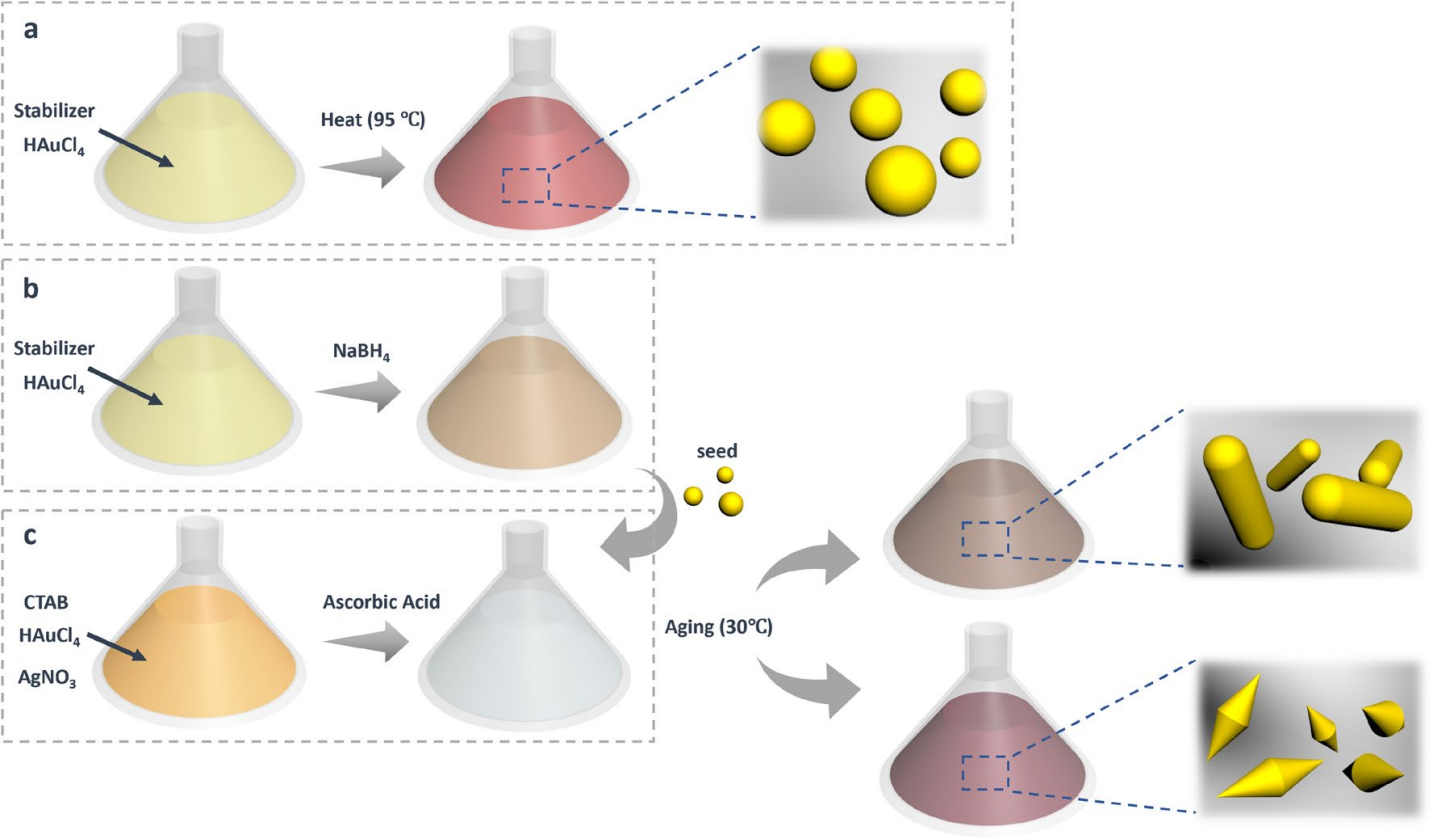
Nanoparticle synthesis flowchart. Gold nanospheres are fabricated by heating the mixture of Au^3+^ and stabilizer. For gold nanorods and nanobipyramids, gold seeds are first generated and then mixed with plate solution. The mixture is aged overnight to obtain gold nanorods and nanobipyramids. (**a**) Synthesis flowchart of gold nanospheres. (**b**) Generation of gold seeds. (**c**) Preparation of plate solution.

The nanoparticle synthesis scheme developed here allowed for fabrication of replicable and consistent nanoparticles in multiple tests. The synthesis flowchart can be found in Fig. 2.

### Nanosphere and nanorod synthesis

In the synthesis of gold nanospheres, 4 mL of 0.2 mg/mL chloroauric acid solution was added to 3 mL of 2 mg/mL sodium citrate solution. The mixed solution was stirred at 400 rpm at 95 ℃ for 20 min. The resulting solution was washed by centrifuge at 4000 rpm for multiple times. The gold nanorods were fabricated using seed-mediated method, which consists two steps: generation of gold seeds and growth of gold around seeds^33^. In order to obtain nanoparticles with specific shape such as nanorods, cetyltrimethylammonium bromide (CTAB) was utilized and acted as a template during the synthesis process and helped to prevent aggregation of gold seeds. In the first step to generate small gold seeds, 0.364 g of CTAB was first dissolved in 5 mL of DI water at 30 ℃ and stirred at 150 rpm for 10 min. Then, 5 mL of 0.2 mg/mL chloroauric acid and 0.6 mL of ice-cold 0.4 mg/mL sodium borohydride was sequentially added to the solution and stirred at 700 rpm for 2 min. The solution turned to brownish yellow after mixing, indicating the formation of small gold seeds. The gold seed solution was kept at room temperature for 2 h before use. During the second step, a plate solution was made to grow the gold seeds to nanorods. To make the plate solution, 0.364 g of CTAB was dissolved in 5 mL DI water, followed by sequential addition of 5 mL of 0.4 mg/mL chloroauric acid, 0.2 mL of 0.7 mg/mL silver nitrate and 70 µL of 13.87 mg/mL ascorbic acid. The plate solution was gently stirred for 1 min before adding 12 µL of the gold seed solution from the first step. The mixed solution was cultured strictly between 27 and 30 ℃ overnight to obtain the gold nanorods.

### Nanobipyramid synthesis and purification

To synthesize gold nanobipyramids, the fabrication process is also based on seed-mediated method^34^. To make the gold seed solution for nanobipyramids, 9.5 mL DI water was mixed with 0.25 mL of 2 mg/mL chloroauric acid solution. Then, 0.25 mL of 3 mg/mL sodium citrate and 0.15 mL of 0.4 mg/mL sodium borohydride was added to the solution sequentially and stirred at 600 rpm for 2 min. The resulting gold seed solution was also kept at room temperature for 2 h before use. A typical plate solution for gold nanobipyramids was made by mixing 9.5 mL DI water, 0.364 g of CTAB, 1 mL of 2 mg/mL chloroauric acid, 0.1 mL of 1.7 mg/mL silver nitrate, 0.2 mL of 1 M hydrochloric acid and 0.08 mL of ascorbic acid in sequence. Finally, the plate solution was mixed with 0.1 mL of the gold seeds solution and kept at room temperature overnight.

As reported in previous literature, during synthesis of the nanobipyramids there existed some gold spherical contaminants and additional purification steps were required^35^. Firstly, the prepared mixture of gold nanobipyramids and nanospheres was washed by centrifuge at 10,000 rpm for 15 min. After resolving the precipitate in 7.5 mL of 25.6 mg/mL CTAB solution, 3.6 mL of 1.7 mg/mL silver nitrate solution and 1.8 mL of 17.6 mg/mL ascorbic acid was added in sequence. The mixed solution was stirred gently for 30 s and then kept in 65 ℃ for 4 h to completely reduce the silver ions. During this period, silver was grown on both gold bipyramids and nanospheres to increase the mass difference between the two shapes of nanoparticles. The resulting gold–silver hybrid nanoparticles were then washed by centrifugation and redispersed in the mixture of 6 mL DI water and 0.109 g of CTAB. After placing the solution in a water bath of 30 ℃ overnight, the silver–gold nanobipyramids were completely settled while the hybrid nanospheres remained in the supernatant. The supernatant was then removed, and the precipitate was resolved in the mixture of 6 mL DI water and 0.091 g of CTAB. After that, 0.1 mL of ammonia hydroxide and 0.1 mL of 5 wt% hydrogen peroxide were sequentially added into the solution and kept for 4 h to etch the silver. The precipitate was then discarded and the resulting solution was washed by centrifuge at 8000 rpm for multiple times to obtain the highly purified gold nanobipyramids. Images of the nanobipyramids before and after purification can be found in the “Supporting Information”.

### Nanoparticle microfluidic printing on chip

Synthesized nanoparticles were dispersed on-chip using a previously published microfluidic printing protocol^1, 2^. Gold nanoparticles in deionized (DI) water were dispersed into the wells of a 96-well plate at a concentration of OD 0.25 (absorbance at peak wavelength). Bare glass slides were functionalized for ten minutes in 10% APTES (3-Aminopropyl triethoxysilane 99%) in anhydrous ethanol before thorough drying. Nanoparticle samples were then printed using a Carterra Continuous Flow Microspotter for 45 min over a glass slide.

### Bulk refractive index testing

One of the simplest sensing applications for plasmonic nanoparticles is an analysis of the shifts in the LSPR spectral peak as the refractive index at the nanoparticle surface changes^14^. In this test, mixtures of water and glycerol were used as a proxy to mimic nanoparticle surface refractive index changes from ctDNA binding. In solution, synthesized gold nanoparticles were centrifuged at 6000 rpm for 8 min to pellet, and the supernatant was removed. The pelleted particles were then incubated in water-glycerol mixtures with varying volume ratios to investigate the response of the surface plasmon peaks to refractive indices (RI). Mixtures of 0% glycerol in water (RI = 1.33), 20% glycerol in water (RI = 1.36), 50% glycerol in water (RI = 1.40), 80% glycerol in water (RI = 1.45), 100% glycerol (RI = 1.47) were used. A full absorbance spectrum of each sample was taken to measure the red shift in the longitudinal peak due to the changing refractive index of the mixture. This spectrum was captured using a Tecan Spark 10 M Microplate reader from 400 to 1000 nm with a step size of 1 nm.

### ctDNA conjugation and testing

The gold nanoparticles dispersed on chip were conjugated with peptide nucleic acid (PNA) probes complimentary to the G12D variant in Exon 2 of the KRAS gene. Only the nanorods and nanobipyramids were tested on-chip because of their promising preliminary results demonstrating the sensitivity of the primary peak. The protocol was based upon a previously published protocol for functionalization of a gold substrate^2, 36^. The gold nanoparticles are incubated with 2.5 mg/mL DSP (dithiobis succinimidyl propionate) in DMSO (dimethyl sulfoxide) for half an hour before a wash with DMSO and then water. Then they are coupled to 1 mg/mL PNA probe in Tris–EDTA buffer for an hour for conjugation, before the sensor is ready to test. These spectra were collected using a FERGIE Integrated Spectrograph (Princeton Instruments) coupled to an optical microscope. A spectrum was taken with both the nanoparticle area and the background in a single measurement, so that the background could be corrected and extinction spectrum calculated 2. These spectra were then processed in MATLAB to calculate the extinction spectrum and peak location. The location of the peak wavelength was determined through a calculation of center of mass of peak boundaries^2, 5^.

### Healthy patient serum testing

The conjugated sensor was tested with synthetic ctDNA spiked into healthy patient serum samples. These samples were pooled discarded, deidentified specimens from the clinical laboratory, with no patient interaction. IRB review was not required for use of these discarded samples because the activity is not research involving human subjects as defined by DHHS and FDA regulations. All methods were carried out in accordance with Dartmouth–Hitchcock Medical Center Guidelines and Regulations.

### Development of electromagnetic simulation

CST Microwave Studio was used to simulate nanoparticles in 3D to determine the effects of geometric and material parameters on the LSPR sensitivity to changes in the refractive index of the surrounding medium. The simulations used a finite integration technique (FIT), and particle geometries were constructed to be consistent with the TEM characterization of the nanoparticles. In order to approximate single particle behavior, 3D nanoparticles were simulated in the center of a cube of medium with boundary conditions examined to show negligible interference at 300 nm in cube length. Far-field monitors were set up to compute an extinction spectrum of incident light upon the particle. Each spectrum was normalized to its peak amplitude for ease of analysis. Each particle was modeled in medium refractive index values of 1.33, 1.40, and 1.47 to simulate particles in water, 50:50 glycerol and water, and pure glycerol, and spectrums were measured between wavelengths of 250–1000 nm. These data were analyzed and compared to the experimentally captured spectra for each nanoparticle geometry.

## Results and discussion

### Nanoparticle characterization and uniformity

**Figure 3:**
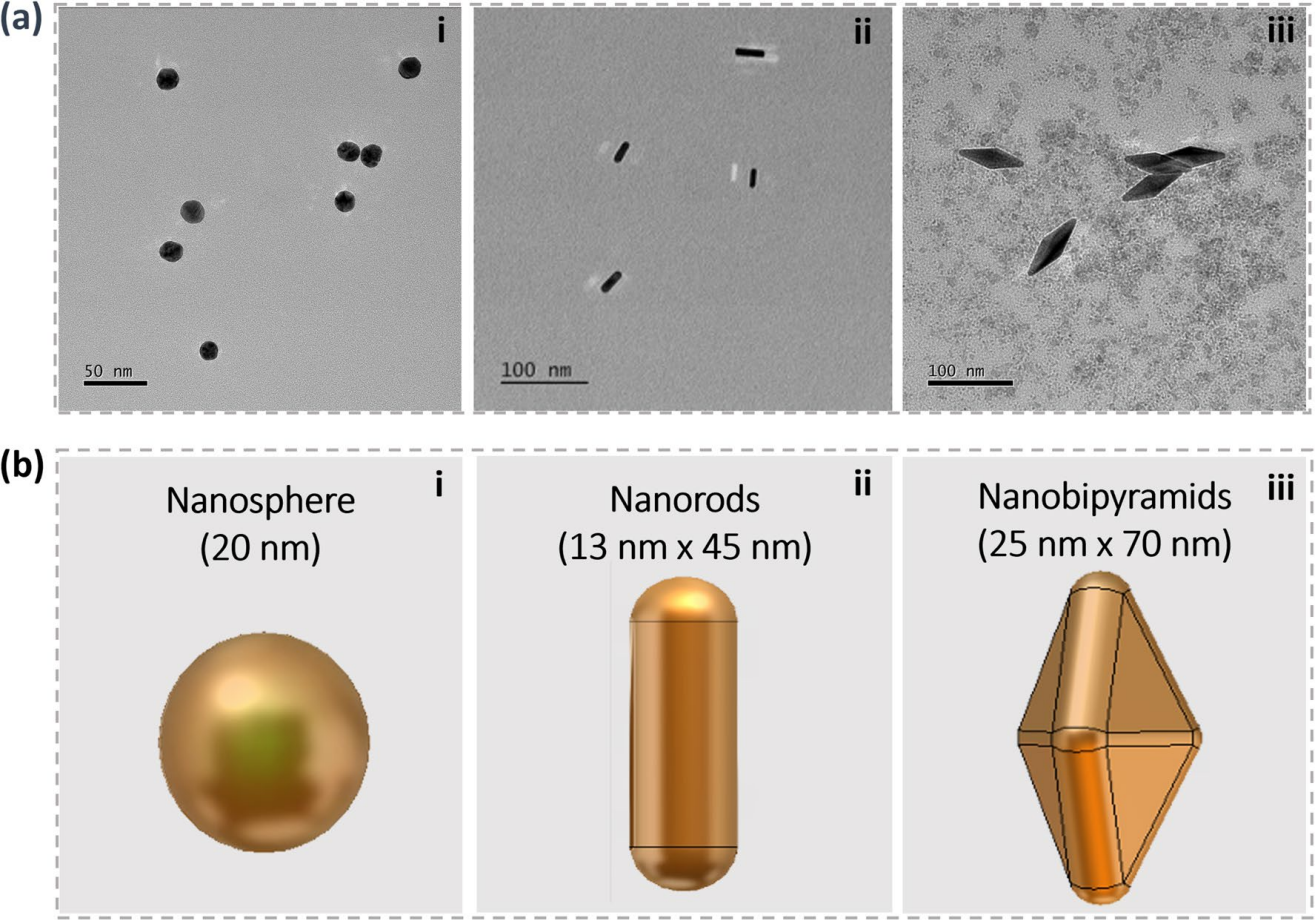
Synthesized nanoparticles and modeled geometries. (**a**) TEM micrographs of the synthesized (i) nanospheres, (ii) nanorods, and (iii) nanobipyramids. (**b**) Dimensions and schematics of the modeled nanoparticles in accordance to the synthesis.

The resulting nanoparticles were characterized using transmission electron microscopy (TEM) to determine their uniformity and size, the results of which for the nanospheres, nanorods, and nanobipyramids are summarized in Fig. 3. The developed synthesis protocol resulted in relatively uniform particles with each of the desired geometries. The dimensions of the particles were collected from ImageJ, resulting in nanospheres measuring about 20 ± 2.8 nm in diameter, nanorods that were 13 ± 2.4 nm in diameter and 45 ± 7.5 nm in length, and nanobipyramids 25 ± 3.2 nm in width and 70 ± 5.2 nm in length. These relatively narrow variances in geometry contribute to the spectral results described later.

### Comparison of resonance spectra

**Figure 4:**
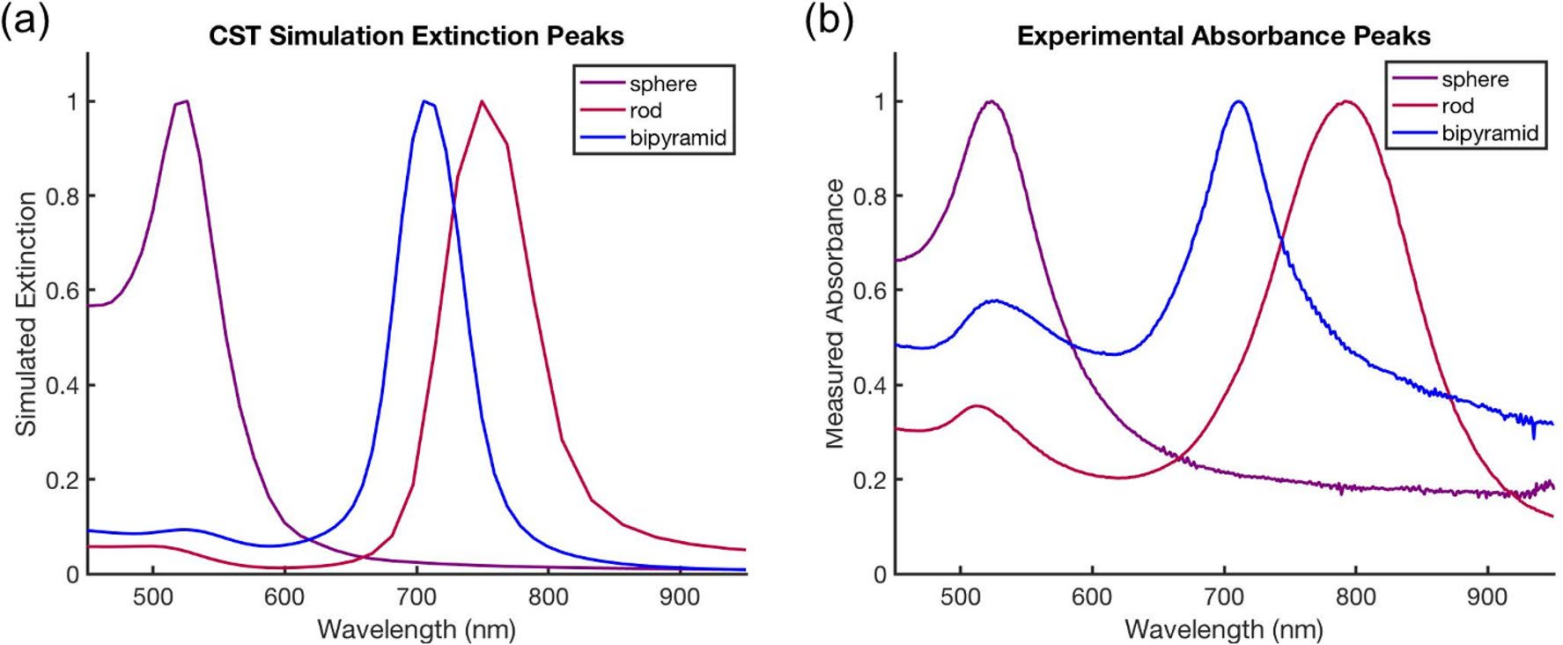
Comparison of simulated and experimental resonance spectra. Extinction peaks calculated from (**a**) simulation demonstrate good agreement with (**b**) experimentally captured spectra. The resonance wavelengths and shapes of peaks are very similar.

After the particles were synthesized with replicable dimensions, the simulated and experimental plasmonic spectra of particles in pure water were compared to determine the level of agreement. These data are summarized in Fig. 4, where one can observe that the spectra qualitatively match up extremely well. In both the simulation and the experiment, there is only one resonance peak for the spheres, and two (a shorter transverse peak and a larger amplitude longitudinal peak) for both the nanorods and the nanobipyramids. There is also significant agreement in terms of resonance location of the plasmonic peaks. The spheres have a resonance around 500 nm, while the primary peak for the nanobipyramids is about 700 nm, and it is closer to 750–800 nm for the nanorods. One thing to be noted is that the full width at half maximum for the experiment is higher than the simulation—in the simulation the peaks are narrower. This is likely due to a slight deviation in the size of the particles when synthesized and experimentally measured whereas the simulation only measures a single particle of fixed size. The background absorbance is also higher in the experimental data compared to the experiment. This is similarly likely due to the fact that the simulation is modeling a single particle in a fixed orientation in water, while the experimental data includes the variation in particle size in a chemical buffer. The qualitative agreement is promising, though, because it shows that the resonance spectrum shape can be predicted by the simulation. For ease of comparison between simulation and experiment, we have normalized the peak intensities of the different particle geometries. However, a deeper dive into these particle geometries would show a greater extinction intensity for the gold nanobipyramids due to their larger size.

### Bulk refractive index sensitivity

After the particles were synthesized and their spectra measured in water, their plasmonic sensitivity was investigated through a bulk refractive index sensitivity test. The nanoparticles were put in contact with water–glycerol mixtures of varying refractive index both experimentally and in simulation. By plotting the refractive index with peak shift, the refractive index sensitivity of the gold nanoparticles to varying refractive index mixtures could be determined. This is a good proxy for plasmonic particle sensitivity and helps to understand nanoparticle geometric effects on overall RI sensitivity. It is expected that these results will qualitatively agree with sensitivity for localized changes in refractive index at the nanoparticle surface, as occurs during biomarker capture.

**Figure 5:**
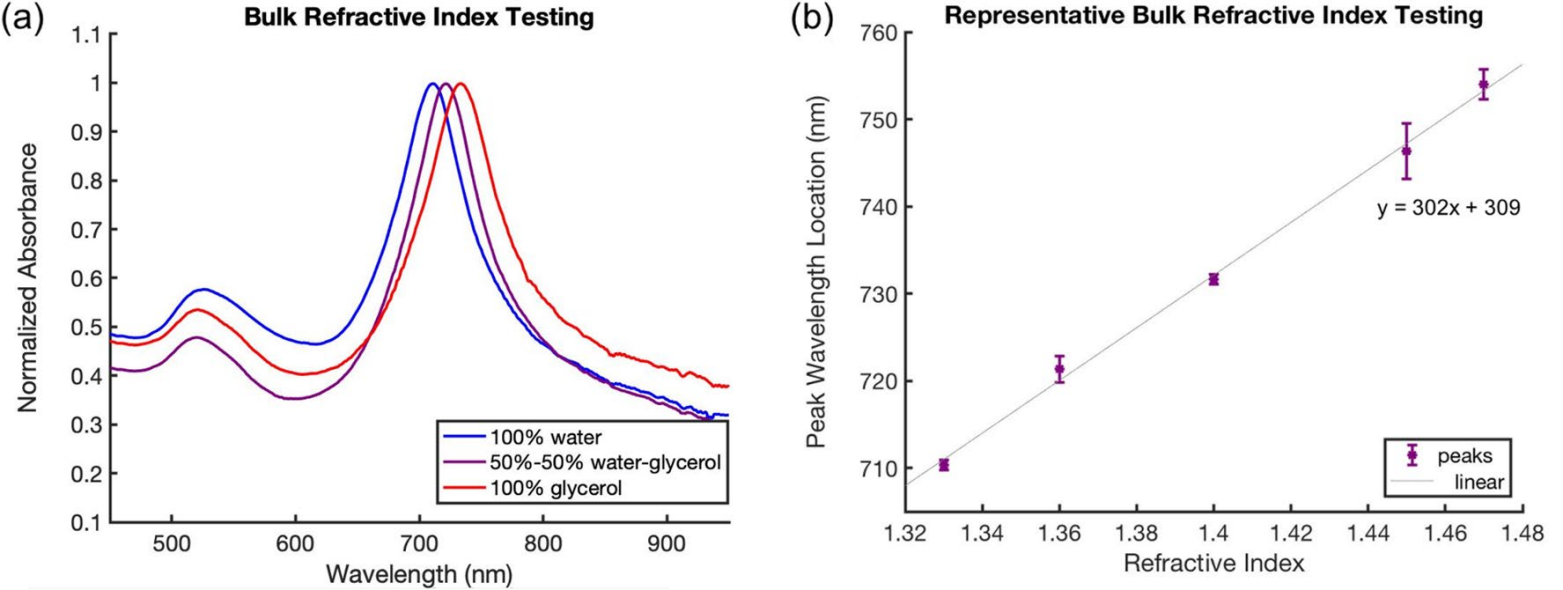
Example of bulk refractive index testing for nanobipyramids. Water-glycerol mixtures were put in contact with nanoparticles—0% glycerol in water (RI = 1.33), 20% glycerol in water (RI = 1.36), 50% glycerol in water (RI = 1.4), 80% glycerol in water (RI = 1.45), 100% glycerol (RI = 1.47). (**a**) Data demonstrating red shifts in resonance peak with increasing glycerol concentration (i.e. increased refractive index). (**b**) Data showing how bulk refractive index sensitivity is calculated based upon the longitudinal peak location. The peak locations are plotted as a function of refractive index. These data for nanobipyramids have a refractive index sensitivity of 302 nm/RIU.

An example of this bulk refractive index procedure can be seen in Fig. 5. As can be observed in inset Fig. 5a, one can observe and quantify the red shift in resonance peak location with increasing bulk refractive index. These peaks can be plotted as a function of refractive index, and a linear relationship determined. The slope of this line is a proxy for how sensitive the particles are to a change in refractive index in their medium. In this example, for the nanobipyramids there is a slope of 302 nm/RIU (refractive index unit).

This experiment was conducted for the nanospheres, nanorods, and nanobipyramids, and was simulated in parallel. There was excellent qualitative agreement between the simulation and experiment, as can be seen in Table 1. As hypothesized, the nanospheres were the least sensitive to changes in RI, the rods were in the middle, and the bipyramids exhibited the highest sensitivity in both experiment and simulation. This is to be expected, because anisotropic structures are generally more sensitive, and sharper corners also improve sensitivity^14, 20–22^. These experimental validations agreed with the theory and simulation, showing that the anisotropic, sharply angled bipyramids had the highest sensitivities. With this data, the rods and bipyramids were used for the ctDNA screening test due to their higher sensitivity and the locations of their resonance peaks within the UV–visible spectrum.

**Table 1:** Simulated and experimental refractive index sensitivity.

	Simulated RI sensivity (nm/RIU)	Experimental RI sensitivity (mm/RIU)
Gold rod (45 nm × 13 nm)	212	228
Gold bipyramid (70 nm × 25 nm)	367	302
Gold sphere (20 nm radius)	73	122

Qualitative agreement can be seen between simulated and experimental refractive index sensitivity.

### ctDNA screening efficacy

After bulk refractive index testing, the particles were dispersed on-chip for conjugation with PNA probes and sequence-specific synthetic ctDNA capture. While the bulk refractive index sensitivity in solution demonstrates the qualitative sensitivity of the particles to binding events, dispersing them on chip allows for rapid sample delivery for use in biosensing. For these ctDNA studies, on-chip particles were used for ease of conjugation and sample delivery to put the particles in contact with synthetic ctDNA sequences of interest.

These experiments were conducted to determine which nanoparticle geometry led to the largest resonance peak shift for known concentrations of synthetic ctDNA. The PNA probe used was complimentary to the G12D variant in the *KRAS* gene, a sequence that is implicated in a number of gastrointestinal cancers, including pancreatic ductal adenocarcinoma. A 100 micron wide, 50 micron deep linear microchannel made of PDMS was used for liquid delivery for all conjugation and patient sensing steps (schematic in “Supporting Information”). The nanoparticles were conjugated on-chip in the microchannel with the PNA probe and successful conjugation was verified based upon resonance peak shift as captured by a spectrophotometer.

**Figure 6:**
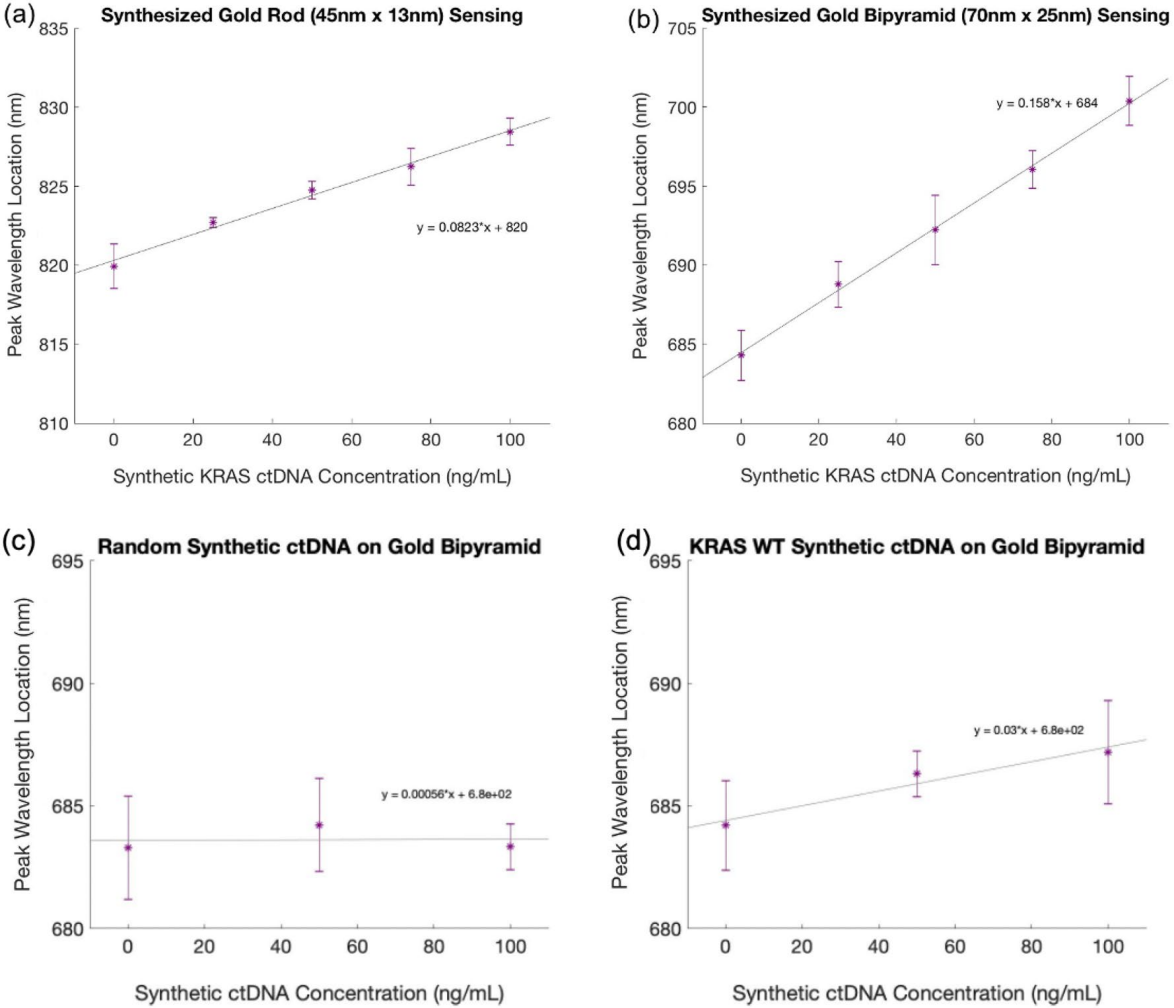
Resonant Shifts for Increasing ctDNA Concentrations using Nanorods and Nanobipyramids. Peak locations for three samples of (**a**) nanorods and (**b**) nanobipyramids at five different synthetic ctDNA concentrations. These data show a linear trend with increasing ctDNA concentration and binding. (**c**) minimal peak shifts when conjugated sensor is put in contact with a random synthetic ctDNA sequence (**d**) very slight peak shifts when sensor is put in contact with the synthetic wild type sequence of KRAS ctDNA.

The location of the plasmonic resonance peak was then measured on three different samples at five different concentrations (0 ng/mL, 25 ng/mL, 50 ng/mL, 75 ng/mL, and 100 ng/mL) of synthetic ctDNA spiked into healthy patient serum samples. The healthy patient samples were discarded, deidentified patient samples that were stored at − 20 °C and used as-is without dilution or filtration. All devices were incubated with the printed nanoparticles for 5 min upon sample delivery. A linear relationship between synthetic ctDNA concentration and peak wavelength location was found for the concentrations tested. This is extremely promising, because it shows that the plasmonic particle sensor has a linear range within the clinically relevant range of ctDNA. These data are shown in Fig. 6 for both the nanorods and the nanobipyramids. There was also minimal nonspecific binding from other blood derivative contents in the patient serum samples.

In order to demonstrate sensor specificity, we also tested the gold nanobipyramids for sequence-specific capture using multiple negative controls. First, we tested a synthetic ctDNA sequence that was randomly generated to have the same GC content as ctDNA, and found no binding to the sensor, as seen in Fig. 6c. This promising result shows that off target nucleic acids in the patient sample do not affect the sensor output. We also tested the specificity of the sensor for detection the G12D point mutation specifically vs. the wild type KRAS sequence (Fig. 6d). Here we showed substantial preference for the mutant compared to the wild type- the sensor only statistically significantly binds the wild type sequence in high concentrations, with an at least five factor preference for the point mutation. This can be seen by comparing the slope in Fig. 6b to that in Fig. 6d. This data shows that if we were to include both a wild type and a mutant probe on the sensor, we would successfully be able to differentiate mutant and wild type binding. Our prior work also highlights additional probe design methodologies to improve the specificity for single point mutations^26^.

These data both show that the synthesized and modeled particles are effective transducers of ctDNA binding for liquid biopsy. Importantly, the data show that the nanobipyramids are significantly more sensitive to the same concentrations of ctDNA binding, as expected from the bulk sensing experiments and the simulation. These binding sensitivities can be calculated from the slope of a linear regression plotted in Fig. 6. For nanorods, this sensitivity is about 0.08 nm/(ng/mL synthetic ctDNA), and for the nanobipyramids, the sensitivity is about 0.16 nm/(ng/mL synthetic ctDNA). This increased sensitivity is almost double for the synthesized nanobipyramids compared to nanorods, which are much more commonly used. The higher binding sensitivity for the nanobipyramids should consequently allow for a significantly lower limit of detection, as smaller changes in peak wavelength can be easily elucidated. This is very encouraging, because it validates our expectation that the angular geometry of the nanobipyramids, along with the larger surface area, lead to improved sensitivity for the plasmonic transduction of clinically relevant biomarkers and concentrations. These data also show a limit of detection based on the spectrometer of ~ 1 ng/mL or in the hundreds of picomolar range. This is approaching the clinically relevant range, with lots of room to optimize nanoparticle geometry and capture chemistry further. Our findings could significantly improve the ability to detect disease earlier and discriminate small changes in concentration for improved diagnostics.

## Conclusion

In this paper, we synthesized three geometries of solid gold nanoparticles and experimentally and theoretically investigated them for applications in plasmonic biosensing of clinically relevant ctDNA. There was excellent qualitative agreement between our electromagnetic simulations and our experimental data: this carried through for both the shape of the resonance spectra and the anticipated refractive index sensitivity. We found that in both simulation and experiment, the nanobipyramids were anticipated to have the highest sensitivity to changes in bulk refractive index, which is a reasonable proxy for surface refractive index changes. This was demonstrated through surface conjugation and synthetic ctDNA experiments, where we found that the nanobipyramids had almost twice the sensitivity to ctDNA binding than the nanorods. This research demonstrates how simulation and experiment can together allow for rational design of nanoparticles for plasmonic biosensing. We hope that this body of work paves the way for future development of nanoparticle geometries and application to ctDNA detection for liquid biopsy.

## Supplementary Information

Supplementary Information.

## Data Availability

The data presented in this study are available upon request. Please contact the corresponding author.
