# Supplementary material for: Rational design of on-chip gold plasmonic nanoparticles towards ctDNA screening
Source: Sci Rep. 2021 Jul 9;11:14185. doi: 10.1038/s41598-021-93207-7 (PMC8270934; doi:10.1038/s41598-021-93207-7)
Supplement: Supplementary file 1 — Supplementary Information. [file 41598_2021_93207_MOESM1_ESM.docx]

**Supporting Information**

# Nanoparticle Purification

As discussed, the nanobipyramid synthesis scheme resulted in small spherical nanoparticle contaminants. **Figure S1** shows before and after TEM images showing the contaminant spheres in the initial sample and the purified nanobipyramids after purification protocol. It can be seen clearly that there are fewer contaminants after purification.

**
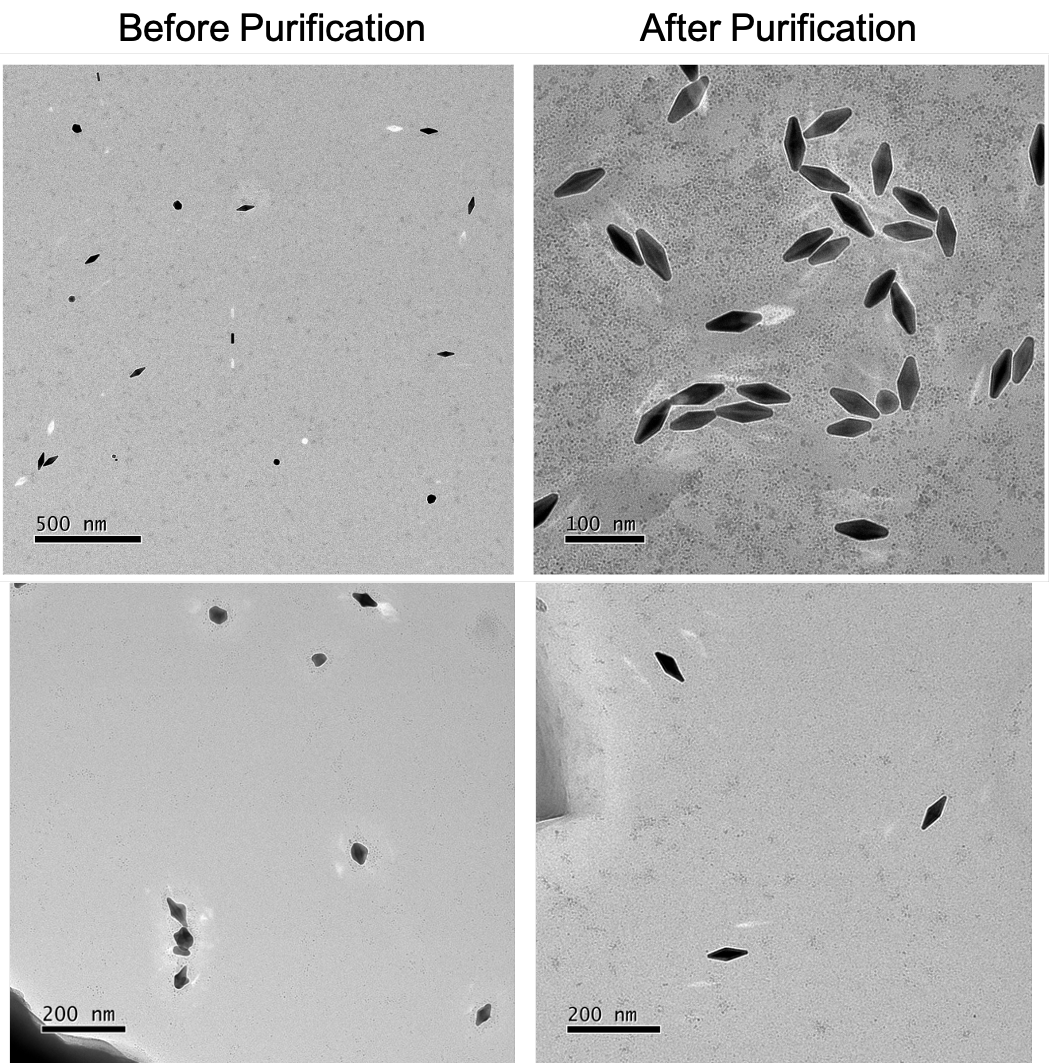
**

**Figure S1.** *TEM characterization of Au bipyramids before and after purification.*  On the left, contaminant spheres can be seen, but on the right we see a purified sample containing only the nanobipyramids.

# ctDNA Screening Details

For demonstration of ctDNA screening, we targeted the G12D mutation in Exon 2 of the KRAS gene. This mutation is relevant to gastrointestinal cancers, among others. The sequence of the PNA probe that was used for capture is: 5’-TAC GCC ATC AGC TCC3’. The sequence of the synthetic duplex DNA used is: 5’ – ACT TGT GGT AGT TGG AGC TGA TGG CGT AGG CAA GAG TGC CT. The random duplex DNA sequence is

5’ AAG ACG TGT ACT GAG GAT CAT ATG ACC ACT TTA TGC CGC TT 3’. The synthetic ctDNA was diluted to the tested concentrations in healthy patient serum samples. An additional study was run to find no shift when the conjugated sensor was put in contact with serum with no present ctDNA.

# Microfluidic Channel

# The following 100 micron wide microchannel was used for all sample delivery in this study. The chip was made out of PDMS using traditional soft lithography techniques.

*
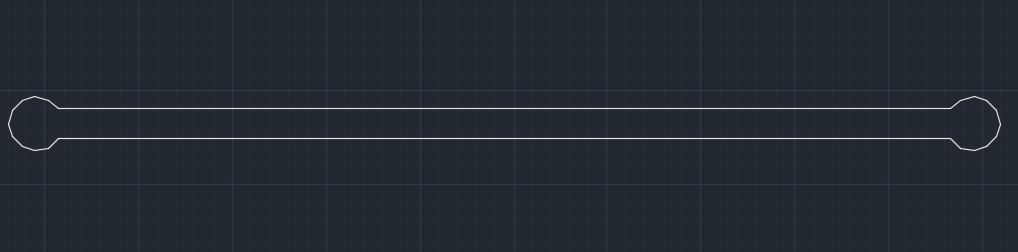
*

**Figure S2.** *Microfluidic Chip Design Schematic from AutoCAD.* Channel width is 100um for scale.

# Particle Synthesis and Replicability

Multiple replicates of the particle fabrication and characterization were carried out, showing consistent results. In these additional studies the geometries and peak locations of the resulting nanoparticles were found to be extremely consistent from batch to batch.

These data demonstrate the replicability of the synthesis and measurement protocols.
